# Treatment of Neurasthenia

**Published:** 1917-07

**Authors:** 


**Treatment of Neurasthenia.** By Dr. PAUL HARTENBERG, translated by Dr. E. PLAYFAIR. Oxford Medical Publications. 1914. Pp. 283. Price 6s. net.

The reader will find in this book a clear and detailed description of the clinical features of Neurasthenia, based upon the author's evidently large experience of the disease. The book follows the ordinary lines. A large part of it is taken up by the chapters on treatment. The author finds in strychnine, given in full doses, preferably subcutaneously and also by the mouth, the most valuable drug. In this respect he differs from those who find that strychnine is not well borne by most neurasthenics. In general the observations on treatment are marked by sound sense, and will be found of great value by anyone who has to deal with these patients, and help him to bring his cases to a successful termination. It is in our opinion a mistake to publish a book of this size without an index.

